# Phyllosphere yeasts rapidly break down biodegradable plastics

**DOI:** 10.1186/2191-0855-1-44

**Published:** 2011-11-29

**Authors:** Hiroko K Kitamoto, Yukiko Shinozaki, Xiao-hong Cao, Tomotake Morita, Masaaki Konishi, Kanako Tago, Hideyuki Kajiwara, Motoo Koitabashi, Shigenobu Yoshida, Takashi Watanabe, Yuka Sameshima-Yamashita, Toshiaki Nakajima-Kambe, Seiya Tsushima

**Affiliations:** 1 National Institute for Agro-Environmental Sciences (NIAES), 3-1-3 Kannondai, Tsukuba, Ibaraki 305-8604 Japan; 2 Research Institute for Innovation in Sustainable Chemistry, National Institute of Advanced Industrial Science and Technology (AIST), Tsukuba Central 5-2, Higashi 1-1-1, Tsukuba, Ibaraki 305-8565, Japan; 3 National Institute of Agrobiological Sciences (NIAS), 2-1-2 Kannondai, Tsukuba, Ibaraki 305-8602, Japan; 4 Graduate School of Life and Environmental Sciences, University of Tsukuba, Tsukuba, 305-8572 Ibaraki, Japan 4; 5 Institute of Biogeosciences, Japan Agency for Marine-Earth Science and Technology (JAMSTEC), 2-15, Natsushima-cho, Yokosuka 237-0061, Japan

**Keywords:** *Pseudozyma*, Biodegradable plastic, Phyllosphere, Yeast

## Abstract

The use of biodegradable plastics can reduce the accumulation of environmentally persistent plastic wastes. The rate of degradation of biodegradable plastics depends on environmental conditions and is highly variable. Techniques for achieving more consistent degradation are needed. However, only a few microorganisms involved in the degradation process have been isolated so far from the environment. Here, we show that *Pseudozyma *spp. yeasts, which are common in the phyllosphere and are easily isolated from plant surfaces, displayed strong degradation activity on films made from poly-butylene succinate or poly-butylene succinate-co-adipate. Strains of *P. antarctica *isolated from leaves and husks of paddy rice displayed strong degradation activity on these films at 30°C. The type strain, *P. antarctica *JCM 10317, and *Pseudozyma *spp. strains from phyllosphere secreted a biodegradable plastic-degrading enzyme with a molecular mass of about 22 kDa. Reliable source of biodegradable plastic-degrading microorganisms are now in our hands.

## Introduction

For the last 60 years, the use of synthetic polymers has grown progressively because of their low cost, reproducibility, and resistance to physical aging and biological attack. However, devices and materials made of synthetic polymers are sometimes used for short-term applications and are subsequently disposed at high rates to the natural environment; ideally such items should be biodegradable so that they do not accumulate in the environment. Biodegradable plastics are a family of polymer products with a molecular structure that is susceptible to biological decomposition into benign or even beneficial products. The environmentally beneficial perception of these materials, and their range of potential applications is expandin: it already extends to composting bags, mulch films, packaging of agricultural supplies, silage wrap, landfill covers, planter boxes, fishing nets, bundling string, seed coatings, and pellet coatings for the delayed release of pesticides, herbicides, and fertilizers (Vert 2005). The rate of degradation of biodegradable plastics in the natural environment is controlled not only by the chemical structure of the plastic, but also by environmental conditions such as temperature, humidity, and nutrient content, all of which influence microbial activity. The rate at which a particular biodegradable plastic will degrade in a given situation is therefore still difficult to determine, and these materials often persist much longer than desired (Sakae et al. 2009). Increased use of biodegradable plastics requires greater reliability of degradation, and one means by which that might be achieved is to better understand the enzymes that efficiently degrade biodegradable plastics and the natural distributions of the microorganisms that produce these enzymes.

The main component of biodegradable mulch films is poly-butylene succinate (PBS). Several biodegradable polymers, such as poly-butylene succinate-co-adipate (PBSA), are added to control mechanical strength (Xu and Guo 2010). Three microorganisms that produce enzymes that degrade solid PBS and PBSA film are the bacterium *Acidovorax delafieldii *strain BS-3, which has been isolated from soil (Uchida et al. 2000), the yeast *Cryptococcus *sp. strain S-2, which is isolated from air or soil (Masaki et al. 2005), and the fungus *Aspergillus oryzae *(Maeda et al. 2005), which is used to produce Japanese rice-wine. However, the efficiency of isolation of microorganisms that degrade solid forms of biodegradable plastic is low. If a reliable source of microorganisms that could degrade biodegradable plastics were found, we could aim to more efficiently recruit such microorganisms to obtain more reliable rates of biodegradable plastic degradation.

Biodegradable plastics are synthesized from the polymerization of diols and dicarboxylic acids by esterification. The aerial parts of higher plants are covered by a continuous extracellular membrane of hydrophobic polymerized lipids called the cuticle. The cuticle is composed of cutin and cuticular wax: cutin is an esterified polymeric network of oxygenated C16 and C18 ω-hydroxylated fatty acids (Heredia 2003), and cuticular wax is composed of hydrocarbons. So both cutin and biodegradable plastics are composed of esterified organic acids that are solid at room temperature. The phyllosphere of healthy plants is normally colonized by bacteria, yeasts, and fungi (Lindow and Brandl 2003). Phyllosphere yeasts are primarily basidiomycete yeasts from the genera *Pseudozyma*, *Cryptococcus*, *Rhodotorula*, and *Sporobolomyces *(Allen 2006). Hydrolytic activity (by proteases, lipases, esterase, pectinases, cellulases, and xylanases) has been observed in yeasts isolated from the phyllosphere (Ruinen 1963, Fonseca and Inácio 2006, Seo et al. 2007).

We observed that the chemical structures of plant surfaces were similar to those of biodegradable plastics; this led us to speculate that the cutinases or lipases from phyllosphere microorganisms might effectively degrade biodegradable plastics. From the phyllosphere, we isolated various species of *Pseudozyma *yeasts that degraded PBS and PBSA films. *Pseudozyma *spp. strains, which were easily isolated from the leaves and husks of paddy rice (*Oryza sativa*) and vegetables, secreted biodegradable plastic-degrading enzymes that degraded PBS or PBSA film to a greater extent than did other microorganisms.

## Materials and Methods

### Substrates and chemicals

To isolate biodegradable plastic-degrading yeasts from the natural environment, we used emulsified PBSA (Bionolle EM-301, average molecular weight 12 to 15 × 10^4^). To evaluate the yeasts' solid polymer-degradation activity we used PBSA film (Bionolle 3001G), and PBS film (Bionolle 1001G), both of which has an average molecular weight 20 to 25 × 10^4 ^and thickness 20 μm. These materials were obtained from Showa Denko K. K. (Tokyo, Japan). The biodegradable plastic-degrading activity of purified enzyme was compared with that of Lipozyme CALB-L (Novozymes A/S; Krogshoejvej, Denmark), a lipase B from *Candida antarctica *produced by genetically modified *Aspergillus niger*. 

### Microorganisms, plants, and media

The microorganisms and rice leaves and seeds used are listed in Tables 1 and 2. Yeast stock cultures were obtained from stock frozen at -80°C and were incubated at 30°C for 3 days on malt-yeast-glucose-peptone (YM) agar medium containing 1% glucose, 0.5% peptone, 0.3% yeast extract, 0.3% malt extract and 1.5% agar. Seed cultures were prepared by inoculating cells grown on YM agar plates into flasks containing fungal minimum medium (FMM) with 4% glucose and incubating them at 30°C on a rotary shaker at 220 rpm for 4 days; the FMM was composed of 0.2% NaNO_3_, 0.02% MgSO_4_, 0.02% KH_2_SO_4_, and 0.1% yeast extract, dissolved in tap water before being autoclaved. For solid cultures, the seed culture (200 μl) was spread on the surfaces of FMM agar plates composed of a bottom layer of FMM-agar medium (15 ml) with no carbon source and an upper layer of 1.5% agar (5 ml) containing a carbon source comprising 1% PBSA emulsion and 1% soybean oil (Wako Chemicals, Osaka, Japan). For liquid cultures, 500-ml Erlenmeyer flasks containing 50 ml of FMM culture medium with one of the following carbon sources: 1% soybean oil, 6% glycerol, or 4% glucose, were inoculated with 500 μl of seed culture and incubated under the same culture conditions as for seed cultures. After cultivation, dry cell weight was determined by collecting the cells from 5 ml of culture broth, washing the pellet with the same amount of deionized water and subsequent drying at 105°C, 17 h.

**Table 1:** Biodegradable plastic-degrading yeasts on rice husks harvested from various areas

JP **no**.	Cultivar name	Origin	Yeast population (cfu/g)		Strain no	Phenotype	Species designation
						
			YM	PBSA			
6775	Shinei	Hokkaido	1 × 10^6^	4 × 10^3^	GB-1	Smooth	*C. flavus*
6918	Akamai	Tohoku	6 × 10^5^	1 × 10^4^	GB-2	Wrinkled	*P. antarctica*
6497	Akinishiki	Niigata	2 × 10^5^	5 × 10^4^	GB-3	Wrinkled	*P. antarctica*
203119^1^	Tankei	Ibaraki	3 × 10^6^	5 × 10^5^	GB-4(0)	Wrinkled	*P. antarctica*
203119^2^	Tankei	Ibaraki	6 × 10^6^	4 × 10^6^	GB-4(1)S	Smooth	*C. laurentii*
				4 × 10^5^	GB-4(1)W	Wrinkled	*P. antarctica*
10995	Nourin18	Kumamoto	8 × 10^5^	6 × 10^5^	GB-5S	Smooth	*C. rajasthanensis*
				3 × 10^5^	GB-5W	Wrinkled	*P. antarctica*
7239	Kanan 2	Taiwan	4 × 10^3^	4 × 10^3^	GB-6	Smooth	*C. laurentii*
5310	Matsumae	Hokkaido	8 × 10^4^	8 × 10^4^	GB-7	Wrinkled	*P. antarctica*
7032	Ouu 52	Akita	8 × 10^5^	8 × 10^5^	GB-8	Wrinkled	*P. antarctica*
7412	Chibanishiki	Chiba	3 × 10^5^	0 × 10^0^	not found		
11295	Saikai 124	Fukuoka	1 × 10^6^	4 × 10^4^	GB-10S	Smooth	*C. rajasthanensis*
				4 × 10^3^	GB-10W	Wrinkled	*P. antarctica*
8955	Kantou 135	Saitama	2 × 10^6^	1 × 10^6^	GB-11S	Smooth	*C. laurentii*
				4 × 10^4^	GB-11W	Wrinkled	*P. antarctica*

All seeds were obtained from National Institute of Agrobiological Sciences (NIAS) Genebank at Tsukua, Japan

Seeds of JP nos. 203119^1^, 5310, 7032, 7412, 11295, and 8955 were harvested in 2000. Those of JP nos. 6775, 6918, 6497, 203119^2^, 10995, and 7239 were harvested in 2005.

**Table 2:** Isolated and stocked *Pseudozyma *spp. strains used in this study

Species	Strain number	Source
*P. antarctica*	JCM10317 ^T^	Lake sediment
*P. rugulosa*	SS1	Pak choi (*Brassica rapa *L. var. *chinensis*)
*P. aphidis*	SS2	Pak choi (*Brassica rapa *L. var. *chinensis*)
*P. aphidis*	SS6	Mizuna (*Brassica rapa *L.var. *nipposinica*)
*P. tsukubaensis*	SS15	Ooba (*Perilla frutescens *Britton var. *crispa *Decne)

JCM: Japan Collection of Microorganisms of the Riken Bio-resource Center, Wako, Japan

^T^: Type strain

### Degradation of plastic films in soil

Soil was obtained from the wheat-cropping fields of NIAES at Tsukuba, Japan. The collected soil was passed through a 2-mm sieve without prior drying. Fresh samples of the sieved soil were used for the analysis after being brought to a moisture content of 50% or 60% of saturated water capacity. Pieces of PBSA film (2 × 2 cm) were packed into sterilized plastic Petri dishes (ϕ90 × D15 mm) with 45 g of the moistened soil (the pieces were sandwiched between a 25-g lower layer and 20-g upper layer) and incubated at 25°C at constant moisture content. Three dishes with seven pieces of film in each were prepared. One piece of film was collected from each dish at intervals of 1 week for 6 weeks, and the degradation ratio of three films, collected each time, was measured by intensity modulation of luminance as follows: An image of the film was scanned with a film scanner and saved in TIFF format. The luminance of the 4-cm^2 ^area of residual film was compared with that of fresh film by using the image-processing software Aquacosmos 2.0 (Hamamatsu Photonics, Shizuoka, Japan). The degradation ratio (%) was then calculated as:

$$\text{Degradat}\text{ion ratio }\left(\%\right)=\frac{\left(\text{luminance of residual film}\right)-\left(\text{luminance of fresh film}\right)}{\left(\text{luminance of background}\right)-\left(\text{luminance of fresh film}\right)}\times 100$$

### Isolation from the phyllosphere of microorganisms that degrade biodegradable plastic emulsion

A sample of about 10 mg of leaves or 40 mg of rice seed husks was beaten in 20 times its weight of 10 mmol sodium phosphate buffer (pH 7.0) with a metallic cone (MC-0212; Yasui Kikai Co., Osaka, Japan) using a multi-beads shocker (model MB501, Yasui Kikai Co.), with the cooling unit at 4°C and shaken at 1500 rpm, in 2 or 3 cycles of 30 s on, 30 s off. The suspension was diluted with the same buffer and spread on an FMM agar plate containing PBSA emulsion and soybean oil with 40 μg ml^-1 ^of chloramphenicol. The plates were incubated at 30°C. A single colony appearing at the center of the clarified PBSA on the plate within 1 week was selected as the strain with the ability to degrade PBSA emulsion.

### Evaluation of degradation activity of yeast strains that degrade biodegradable plastic film

Seed culture (500 μl) of yeast strains selected for their ability to degrade PBSA emulsion on agar plates was spread onto 9 cm-diameter FMM agar plates containing 1% PBSA emulsion and 1% soybean oil in the top layer. After incubation of the inoculated plates at 30°C overnight, squares of the target biodegradable plastic film (2 × 2 cm) were mounted on the surface of the yeast lawn of the plate. After incubation of the plate at 30°C, the films were collected at designated time intervals. The degradation ratio of films was measured as above.

### Identification of microorganisms that degrade biodegradable plastic

The microorganisms isolated from phyllosphere materials were identified by rDNA sequence homology by BLAST search in the DNA Data Bank of Japan (DDBJ). rDNA sequences were obtained as follows. To extract the genomic DNA of the isolated microorganisms, cells were suspended in 100 μl each of Tris-EDTA (TE)-saturated phenol and TE buffer, and were disrupted by beating with zirconia beads (Wen et al. 2005). The TE layer was used as a DNA template to amplify the rDNA sequence with NL1 (5'-gcatatcaataagcggaggaaaag-3') and NL4 (5'-ggtccgtgtttcaagacgg-3') as primers. The amplified DNA fragments were purified with a GeneElute PCR Clean-Up Kit (Sigma-Aldrich, St Louis, MO) and sequenced with the same primers. All DNA sequences were determined by means of a 3100 Genetic Analyzer (Applied Biosystems, Foster City, CA) using a BigDye Terminator v.3.1 cycle sequencing kit (Applied Biosystems). The nucleotide sequence of the rDNA was compared with those in the DDBJ by using the Blast search with nucleotide sequence database.

### Assay for activity of enzymes that degrade emulsified biodegradable plastic

The degradation activity of enzymes on biodegradable plastic was measured in a glass test tube (10 mm internal diameter) by spectrophotometry. PBSA emulsion was suspended in 2 ml of 10 mmol Tris-HCl buffer (pH 6.8) containing either supernatant from the culture medium or purified enzyme solution. The percent transmittance of biodegradable plastic emulsion at a wavelength of 660 nm was measured as the reduction in absorbance. One unit of PBSA-degradation activity was defined as a 1-U decrease in absorbance at 660 nm min-1. Assays for the esterase activity was performed in 96-well microplates, 70 μl of 50 mM Tris-HCl (pH8.0) and 20 μl of 1 mM of *para*-nitrophenyl (*p*NP)-butyrate or *p*NP-palmitate (Sigma-Aldrich) in DMSO were mixed in a well. Reactions were initiated by the addition of 10 μl of PaE (final concentration of 34 nM), and the reaction was carried out at 30°C for 5 min, then the absorbance at 400 nm was measured by multi-spectrophotometer (Dainippon, Osaka, Japan). The absorbance of each substrate in the buffer without enzyme was subtracted as a blank to take into account the substrate's autohydrolysis in the solution. One unit of esterase activity was defined as the release of one micromole of *p*NP per minute.

### Purification of enzymes that degrade biodegradable plastic

Liquid culture medium (500 ml of FMM with glycerol) of *P. antarctica *JCM 10317 in which emulsion-degrading activity had been confirmed was centrifuged at 7000 × *g *for 15 min and the supernatant was passed through a paper filter (Advantec No. 2, Toyo Roshi Kaisha, Tokyo, Japan). Ammonium sulfate powder was stirred into the filtrate to 50% saturation at 4°C, and the mixture was centrifuged at 20 000 × *g *for 15 min. The precipitate was suspended in 20 mmol Tris-HCl buffer (pH 6.8) and dialyzed against the same buffer. Ion exchange chromatography of the enzyme was performed in accordance with a method for the purification of lipase and cutinase (Kolattukudy et al. 1981, Kamini et al. 2000, Kakugawa et al. 2001, Akutsu-Shigeno et al. 2003) as follows. The crude enzyme was applied to a DEAE-Sepharose Fast Flow column (GE Healthcare Bio-Sciences, Buckinghamshire, England). The non-absorbed fraction recovered after passing through the column was applied to an SP-Sepharose Fast Flow column (GE Healthcare Bio-Sciences). After the column had been washed with the same buffer, the protein was eluted by addition of the same buffer containing 0.05 mol l^-1 ^NaCl. The enzyme solution was concentrated by ultrafiltration (Centricut 10,000, Kurabo, Osaka, Japan) and applied to a gel-filtration column (TSK-gel G3000SW_XL_, Tosoh, Tokyo, Japan) with a running buffer of 50 mmol Tris-HCl (pH 6.8) containing 0.3 mol l^-1 ^NaCl. The enzymatically active fractions were collected. The protein concentration was determined with a protein assay kit (Bio-Rad Laboratories, Hercules, CA) according to the manufacturer's instructions, by using bovine serum albumin (Sigma-Aldrich) as the standard. We named the enzyme that degrades biodegradable plastic from *P. antarctica*, as PaE.

### Protein analysis of biodegradable plastic-degrading enzyme

During the protein purification steps of the column chromatography, the solutions containing enzymatically active protein were separated by sodium dodecyl sulphate - polyacrylamide gel electrophoresis (SDS-PAGE) according to the method of Laemmli (1970), using a 14.1% polyacrylamide slab gel. The gel was stained with Coomassie blue (CBB; PhastGel Blue R, GE Healthcare, Little Chalfont, England). Protein purity was confirmed by silver staining (Daiichi Kagaku Yakuhin, Tokyo, Japan) of the SDS-PAGE, using a 12% gel. A single protein spot, stained with CBB, was subjected to mass spectrometry by the method described by Shevchenko et al. (1996). Rabbit PaE polyclonal antibody was prepared from the single protein spot. The presence of PaE homolog in the culture broths from various *Pseudozyma *spp. was detected by using Western blot analysis with anti-PaE. Trichloroacetate (TCA)-precipitated proteins from 100 μl of each culture broth were separated by means of SDS-PAGE, as above. After the transfer of the proteins to a polyvinylidene difluoride membrane (Fluorotrans, Pall, Port Washington, NY), PaE was detected according to the manufacturer's instructions of ECL detection system (GE Healthcare). The membrane was blocked with 5% skim-milk in phosphate buffered saline with Tween 20 (PBST) and incubated overnight with PaE antiserum (1/3000) at 4°C. It was then washed with PBST and incubated with horseradish peroxidase-linked goat anti-rabbit IgG (BioRad) and examined with an ECL detection system. The E-PAGE MagicMark Unstained Protein Standard (Invitrogen, Carlsbad, California) was used for the molecular weight estimation of proteins after western blotting.

## Results

### Typical rates of degradation of biodegradable plastic in soil

**Figure 1:**
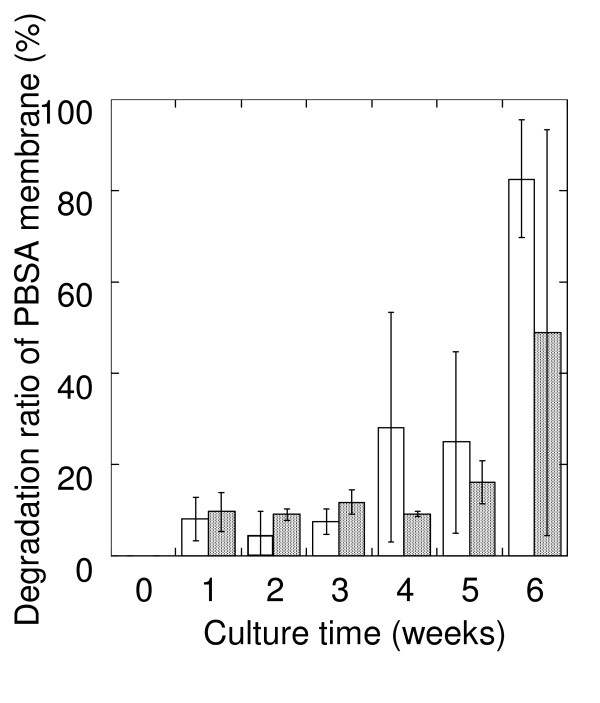
**Degradation of PBSA mulch film in soil**. Degradation of 2 × 2 cm squares of PBSA mulch film packed in soil at water contents of 50% (hatched bar) and 60% (open bar) and incubated at 25°C.

To determine the typical rate of biodegradation of biodegradable plastic, we incubated mulch film made from PBSA (2 × 2 cm squares) in soil obtained from fields. After 4 weeks of incubation at 25°C, the film had decomposed by 28.2% (SD = 25.2) at a soil moisture content of 60%, and 9.1% (SD = 0.65) at a moisture content of 50%. After 5 and 6 weeks, the degradation rates at a soil moisture content of 50% are 19.8% (SD = 4.74) and 48.9% (SD = 44.3) (Figure 1).

### Isolation of phyllosphere yeast strains capable of degrading biodegradable plastic film

**Figure 2:**
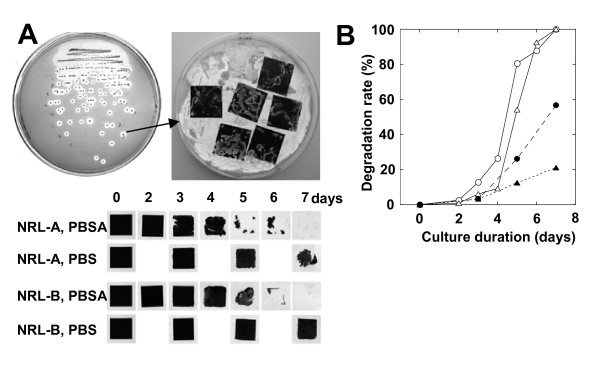
**Screening and evaluation of biodegradable plastic degrading yeast from phyllosphere**. (A) Screening method and degradation of 2 × 2-cm squares of PBSA and PBS mulch film at 30°C by yeasts (NRL-A and NRL-B) isolated from two rice leaves. (B) Quantification of film degradation rates of PBSA film by strain NRL-A (*open circle*), NRL-B (*open triangle*), and degradation rates of PBS film by NRL-A (*closed circle*), NRL-B (*closed triangle*).

We observed that the chemical structures of plant surfaces are similar to those of biodegradable plastics, which led us to determine whether the microflora of plant surfaces might produce enzymes with activity against biodegradable plastics. Several strains of phyllosphere yeasts are reported to produce lipases (Ruinen 1963, Fonseca and Inácio 2006, Seo et al. 2007). Therefore, we speculated that the lipases from phyllosphere microorganisms might effectively degrade biodegradable plastics. For isolation of such yeasts, we used FMM agar plates containing oil and an emulsified biodegradable plastic (i.e. PBSA) in the upper layer, along with nutrients suited to the isolation of yeast. Yeast colonies that could assimilate PBSA emulsion or oil by producing lipase or esterase were expected to grow on this medium and to become surrounded by a clear zone if the enzyme degraded the PBSA emulsion (Figure 2A). Such yeasts were isolated from two leaves of paddy rice (Figure 2A, B). The colony surfaces of yeast on each selection agar plate are uniform. Both strains were identified as *P. antarctica*, and they were named NRL-A and NRL-B. At the second screening, squares of the target biodegradable plastic film (2 × 2 cm) were mounted on the surface of the yeast lawn of the plate, and observed the degradation rate as described in material and methods. Both strains degraded PBS and PBSA films on the same agar plate. As previously reported (Uchida et al. 2000, Maeda et al. 2005, Masaki et al. 2005), PBSA film was easier to biodegrade than PBS film.

### Populations and characterization of yeasts that were found on rice husks and degraded biodegradable plastic

**Figure 3:**
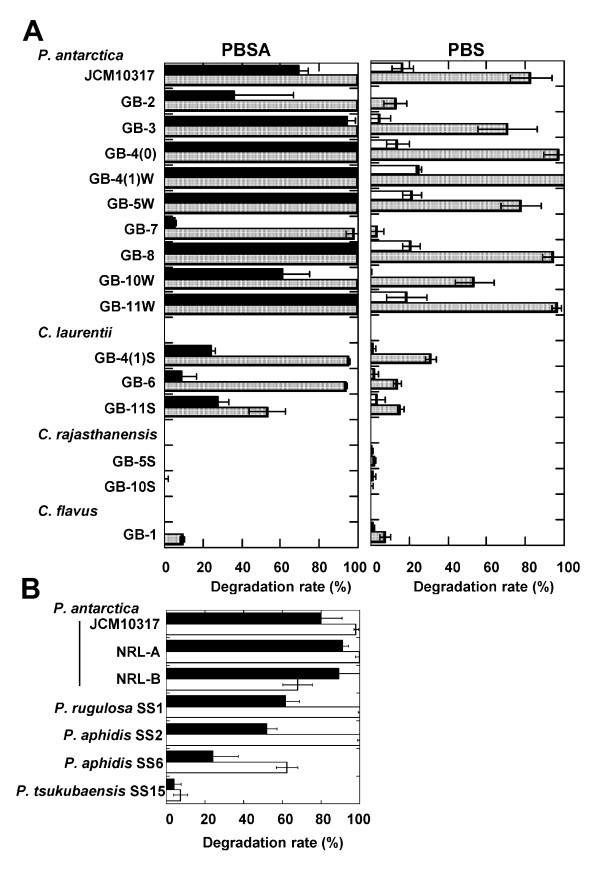
**PBSA and PBS film-degradation activity of various species of phyllosphere yeasts**. (A) PBSA and PBS film-degradation activity of yeast strains isolated from the husks of rice cultivated in various areas. Degradation rate of films after cultivation for 2 days (*closed bar*), 3 days (*open bar*), and 5 days (*hatched bar*), and (B) PBSA (closed bar, at 2 days) and PBS film (open bar, at 5 days)-degradation activity of various species of *Pseudozyma *collected from the leaves of rice and vegetables. Values are expressed as the mean (SD) at n = 3.

We next examined the distribution of biodegradable plastic-degrading yeasts in the phyllosphere. We analyzed the populations of these yeast strains on the 12 stocked seed husks of 11 rice cultivars collected from various locations in Japan and Taiwan (Table 1). The yeast populations on the rice husks ranged from 4 × 10^3 ^to 6 × 10^6 ^cfu/g. The populations of PBSA emulsion-degradable yeasts ranged from 4 × 10^3 ^to 4 × 10^6 ^cfu/g in 10 seed-husk collections. PBSA emulsion-degrading clones therefore constituted 2% to 100% of the yeast populations. All PBSA emulsion-degrading colonies observed in every culture have the same morphological characteristics (wrinkled or smooth), but those in Tankei 9001 (harvested in 2005), Nourin 18, Saikai 124, and Kantou 135 showed two different colony surface phenotype (wrinkled or smooth). We were unable to identify any yeast colonies in the culture from Chibanishiki because of heavy mold contamination. For detailed analysis, we picked one PBSA emulsion-degrading colony of uniform surface phenotype from each culture. All clones isolated from the wrinkled-surface colonies were identified as *P. antarctica*, and all of those from the smooth-surface colonies were identified as *Cryptococcus *spp. We tested the PBS and PBSA film-biodegradation activity of the isolated strains on FMM agar plates containing oil and PBSA emulsion (Figure 3A). Strains of *P. antarctica *are isolated from 9 of 12 stocked rice husks. And all strains of *P. antarctica *quickly degraded both PBSA and PBS films on the agar plates. Strains of *C. laurentii *also degraded PBSA film well but were slow to degrade PBS film. A strain of *C. flavus *slowly degraded PBSA and PBS films, but two strains of *C. rajasthanensis *did not degrade either type of film. Thus, of the 17 strains that were isolated from rice leaves or husks and degraded PBSA emulsion, 15 also degraded solid film. The type strain *P. antarctica *JCM 10317 also degraded both PBSA and PBS films (Figure 3A, B). Therefore, strains of *P. antarctica *are common colonizers of the surface of rice leaves and husks and can degrade biodegradable plastic.

### Biodegradable plastic-degrading activity of various species of *Pseudozyma*

We speculated that species of the genus *Pseudozyma *other than *P. antarctica *would be able to degrade biodegradable plastics. We tested the film degradation activities of *Pseudozyma *spp. strains isolated from the leaves of vegetables; these were classified as *P. rugulosa *SS1, *P. aphidis *SS2, SS6, and *P. tsukubaensis *SS15 and were stocked at National Institute of Advanced Industrial Science and Technology (AIST) (Table 2). All of the tested strains degraded both PBSA and PBS films (Figure 3B).

### Culture conditions for inducing degradation of biodegradable plastics

**Figure 4:**
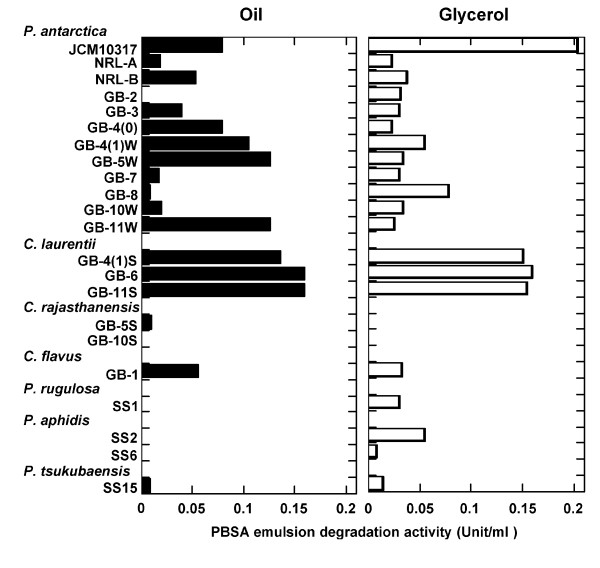
**Degradation of PBSA emulsion by culture broths of yeast strains isolated from phyllosphere**. Yeast strains were cultivated in FMM liquid medium with soybean oil or glycerol as a carbon source. After 3 days' cultivation, PBSA emulsion degradation activity was measured.

The next step was to analyze the culture conditions under which the isolated strains produced enzymes that could degrade biodegradable plastics. We analyzed the degradation of emulsified PBSA in FMM liquid culture medium to which soybean oil or glycerol had been added as a carbon source (Figure 4). Strains of *P. antarctica, C. laurentii*, and *C. flavus *degraded the PBSA emulsion in the presence of either carbon source. We did not detect PBSA emulsion-degrading activity in any of the culture broths of *C. rajasthanensis *strains. PBSA emulsion degradation activities were not detected on the vegetable strains of *P. rugulosa*, *P. aphidis *and *P. tsukubaensis *in FMM medium with oil, but weak activities were observed in FMM medium with glycerol.

Because *P. antarctica *JCM10317 strongly degraded both PBS and PBSA films and PBSA emulsion, we further analyzed the conditions under which degradation by this strain was induced. When the medium contained glucose, no degradation of PBSA was detected until after 3 days of incubation, by which time all the glucose had been consumed; PBSA degradation activity was then initiated (data not shown). The dry cell weight of the culture broth after 3 days of incubation in FMM with glucose, oil, or glycerol is 13.3, 8.4, and 7.7 mg/ml, respectively. So, the enzyme activity is not dependent on growth rate. These results suggested that production of the enzymes involved in degrading biodegradable plastic was suppressed by the presence of glucose as a carbon source in the medium.

### Purification and characterization of enzymes that degrade biodegradable plastics

**Figure 5:**
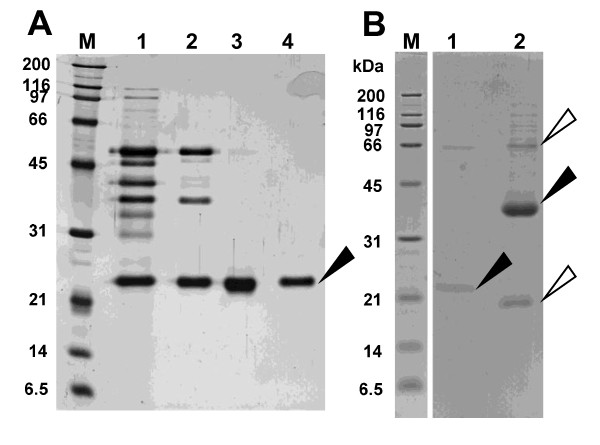
**SDS-PAGE of biodegradable plastic-degrading enzyme (PaE) from *Pseudozyma antarctica *JCM 10317 and a commercially available lipase, CALB-L**. (A) Purification of PaE. **M **Molecular mass standards; **1 **Ammoniun sulfate precipitate; Fractions of **2 **passed through the DEAE-Sepharose column; **3 **eluated from SP-Sepharose column; **4 **Gel filtration. SDS-PAGE was performed using 12% gel and detected by silver staining (B) Purified PaE and CALB-L. **M **Molecular mass standards; **1 **purified PaE (0.25 μg); **2 **CALB-L (25 μg). SDS-PAGE was performed using 14.1% gel and detected by CBB. The black arrow in the PaE lane shows the PaE isolated from the culture medium. The black arrow in the CALB-L lane indicates lipase B. The white arrows indicate impurities.

The strain *P. antarctica *JCM10317 is synonymous with *Candida antarctica *CBS 5955, and known to produce two lipases, lipase A (43 kDa) and lipase B (33 kDa), on culture media containing oil (Ishii 1988). As the culture conditions required for producing lipases and PaE were identical, we postulated that these common lipases from the strain could degrade biodegradable plastic. However, from a culture medium of *P. antarctica *JCM10317, we purified an enzyme that degraded biodegradable plastic (PaE) and had a molecular weight of about 22 kDa (Table 3, Figure 5). At pH 6.8, the relative degradation activity of commercially available lipase B (CALB-L) on PBSA emulsion was about 1/6500th that of PaE. We confirmed by MS fingerprinting of PaE and lipase B that PaE was a different protein from lipases A and B (data not shown). Esterase activites of PaE against *p*NP-butyrate and *p*NP-palmitate are 715.33 ± 124.44 U/mg PaE and 194.72 ± 36.11 U/mg PaE, respectively, showing that PaE is an esterase with wide spectrum to degrade esterified bond of long-chain fatty acids.

**Table 3:** Purification of biodegradable plastic-degrading enzyme from *P. antarctica *JCM10317

Source of fraction	Total protein (mg)	Total activity (U)	Specific activity (U/mg)	Yield (%)
Culture filtrate	23.306	28.96	1.24	100.0
Ammonium sulfate precipitation	0.422	3.42	8.11	11.83
Ion exchange chromatography	0.014	2.73	198.35	9.44
Gel filtration	0.003	1.34	398.16	4.61

**Figure 6:**
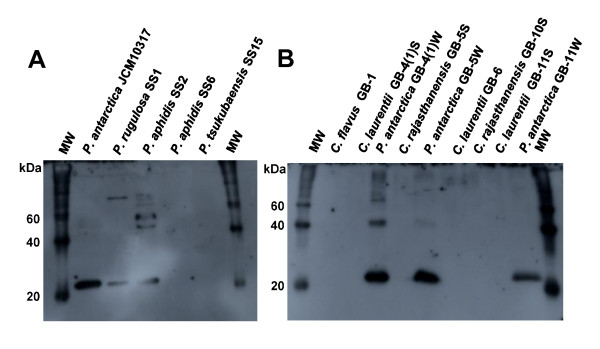
**Immunological identification of biodegradable plastic-degrading enzymes of phyllosphere yeasts**. Yeasts from vegetables (A) and rice husks (B) were cultivated in FMM liquid medium with glycerol for 3 days. TCA-precipitated culture broth (100 μl) was separated by using SDS gel electrophoresis and analyzed by use of Western blotting with anti-PaE. **M **Molecular mass standards.

Rabbit PaE polyclonal antibody was prepared by Tanpaku Seiseikogyo (Isezaki, Japan) from the single protein spot, and confirmed the specificity of antibody to PaE by western blotting (Figure 6A). The immunological identity of biodegradable plastic-degrading enzymes among yeasts isolated from rice husks was analyzed by Western blotting of samples cultured for 3 days in FMM-glycerol liquid medium and incubated with anti-PaE. A hybridized band with uniform mobility was detected from the culture broths of all *P. antarctica *strains, *P. rugulosa *SS1, and *P. aphidis *SS2 but no band was detected from the culture broths of *C. laurentii *(Figure 6A, B) even the PBSA emulsion degradation activities were detected (Figure 4).

## Discussion

The rate of degradation of biodegradable plastics is controlled not only by their chemical structure but also by environmental conditions such as temperature, humidity and nutrient content. For example, after 4 weeks' incubation at 25°C in soil from a vegetable field, mulch film made from PBSA had decomposed by 28.2% (SD = 25.2) at a soil moisture content of 60% but only by 9.1% (SD = 0.65) at a moisture content of 50% (Figure 1). However, under the uniform environmental conditions, the degradation speed is quite variable in soil after average degradation rate over 20%. For example, after 5 weeks' incubation at a soil moisture content of 50%, film had decomposed by 19.8% (SD = 4.74) but by 48.9% (SD = 44.3) at 6 weeks' incubation. This shows there is considerable variability in biodegradation rates under controlled conditions by various unknown factors, and to improve the degradation reliability of biodegradable plastics we need to better understand the enzymes involved and the natural distributions of the microorganisms that produce those enzymes. However, we simply do not know where active populations of such degradative microorganisms naturally occur. Therefore, the isolation efficiency of microorganisms that degrade biodegradable plastic is low.

Microorganisms that inhabit the surfaces of natural materials can often degrade those materials in order to adhere strongly to the surface or take in the degradation products as nutrients. We observed that the structures of biodegradable plastic and cutin were similar in that both were made from esterified organic acids in solid form at room temperature. In light of the lipase and esterase activities of phyllosphere yeasts, we first attempted to isolate phyllosphere yeasts that could degrade emulsified biodegradable plastic. As strains of *Fusarium *sp. and *Pseudozyma *spp. produce lipase on media containing natural oils as the sole carbon sources (Ishii 1988, Feng et al. 2005), we speculated that the lipase or esterase from phyllosphere microorganisms might effectively degrade biodegradable plastics.

We were successful in isolating yeasts that degrade biodegradable plastics from the phyllosphere on minimum medium agar plates containing oil and emulsified PBSA. We found that the two yeasts isolated from two leaves of paddy rice on these plates could degrade biodegradable plastic emulsion (Figure 2A, B), and from the seed husks of 11 of 12 rice cultivars collected from various locations (Table 1). The isolation efficiency is 2 to 100% of phyllosphere yeast population. Of the 17 strains that were isolated from rice leaves or husks and degraded PBSA emulsion, 15 also degraded solid film; in contrast, when we isolated biodegradable plastic-degrading bacteria from soil or sediment (Uchida et al. 2000), only 1% to 2% of the bacteria that degraded the PBSA emulsion degraded solid film (unpublished data). These results show that the populations of biodegradable plastic-degrading yeasts in the phyllosphere were quite high in the natural environment. Strains of *P. antarctica *with strong PBS and PBSA film degradation activities are isolated among 9 of 12 rice cultivars. These results showed that strains of *P. antarctica *are common colonisers of the surfaces of rice leaves and husks and are capable of degrading biodegradable plastics. Yeast strains of the genus *Pseudozyma *are often isolated from various plant surfaces (Allen 2006). We therefore speculated that species of the genus *Pseudozyma *other than *P. antarctica *may be able to degrade biodegradable plastics. All the tested strains of *Pseudozyma *spp., isolated from phyllosphere and stocked in our collection, degraded PBS and PBSA films (Figure 3B). Of the tested strains, the type strain *P*. *antarctica *JCM10317 demonstrated the strongest degradation activity on PBS and PBSA mulch film (Figure 3A, B). A strain of *Candida antarctica *CBS 5955 (a synonym of *P. antarctica *JCM 10317) is known to produce two lipases, lipase A (43 kDa) and lipase B (33 kDa), on culture media containing oil (Ishii 1988). Thus we speculated such lipases may degrade biodegradable plastics. However, from a culture medium of *P. antarctica *JCM10317, we purified an enzyme that degraded biodegradable plastic (PaE) and had a molecular weight of about 22 kDa (Figure 5). The relative degradation activity of PBSA emulsion by lipase B (CALB-L) was about 1/6500th of that of PaE. We have confirmed that PaE was a different protein from lipase A and B. Furthermore, we have observed that all isolated *Pseudozyma *spp. strains, which showed biodegradable plastic film degradation ability, secreted immunologically identical biodegradable plastic-degrading enzyme; but there may be the structural differences in enzymes from *Cryptococcus *spp. between PaE (Figure 6). These results demonstrated that the strains of the genus *Pseudozyma *isolated in this study degrade biodegradable plastics using a novel enzyme. In order to accelerate the degradation of biodegradable plastic wastes, we therefore propose that they be treated with these phyllosphere yeasts or their enzymes. Further studies should be conducted to characterize these yeasts and their enzymes as well as to develop practical methods for utilizing them.

## Competing interests

The authors declare that they have no competing interests.
